# Physico-Chemical Characteristics of Lipoplexes Influence Cell Uptake Mechanisms and Transfection Efficacy

**DOI:** 10.1371/journal.pone.0006058

**Published:** 2009-06-26

**Authors:** Sarah Resina, Paul Prevot, Alain R. Thierry

**Affiliations:** 1 Laboratoire de Dynamique des Interactions Membranaires Normales et Pathologiques (DIMNP), Département de Défenses Antivirales et Antitumorales - UMR 5235 - Université de Montpellier II, Montpellier, France; 2 Modélisation et Ingénierie des Systèmes Complexes Biologiques pour le Diagnostic (SysDiag) – UMR 3145 CNRS/Bio-Rad - Cap Delta, Montpellier, France; University of Southampron, United Kingdom

## Abstract

**Background:**

Formulation of DNA/cationic lipid complexes (lipoplexes) designed for nucleic acid delivery mostly results in positively charged particles which are thought to enter cells by endocytosis. We recently developed a lipoplex formulation called Neutraplex that allows preparation of both cationic and anionic stable complexes with similar lipid content and ultrastructure.

**Methodology/Principal Findings:**

To assess whether the global net charge could influence cell uptake and activity of the transported oligonucleotides (ON), we prepared lipoplexes with positive and negative charges and compared: (i) their physicochemical properties by zeta potential analysis and dynamic light scattering, (ii) their cell uptake by fluorescence microscopy and flow cytometry, and (iii) the biological activity of the transported ON using a splicing correction assay. We show that positively or negatively charged lipoplexes enter cells cells using both temperature-dependent and -independent uptake mechanisms. Specifically, positively charged lipoplexes predominantly use a temperature-dependent transport when cells are incubated OptiMEM medium. Anionic lipoplexes favour an energy-independent transport and show higher ON activity than cationic lipoplexes in presence of serum. However, lipoplexes with high positive global net charge and OptiMEM medium give the highest uptake and ON activity levels.

**Conclusions:**

These findings suggest that, in addition to endocytosis, lipoplexes may enter cell via a temperature-independent mechanism, which could be mediated by lipid mixing. Such characteristics might arise from the specific lipoplex ultrastructure and should be taken into consideration when developing lipoplexes designed for *in vivo* or *ex vivo* nucleic acid transfer.

## Introduction

Non-viral DNA delivery systems have been developed to facilitate gene entry into mammalian cells. Among the polycharged vectors currently used, DNA/cationic lipid complexes (lipoplexes) [1] seem promising candidates since they deliver nucleic acids efficiently both *in vitro* and *in vivo* [1], [2], [3], [4]. Indeed, various systems are now commercially available for lipoplex-mediated transfection (lipofection) of cultured cells and 7.1% of the current gene therapy clinical trials concern the evaluation of lipoplex formulations to deliver genes, active molecules or drugs (www.wiley.co.uk/genetherapy/clinical/). However, despite the great success for *in vitro* transfection, cationic lipoplexes often exhibit significant drawbacks when used for *in vivo* delivery. Indeed, plasma proteins with anionic charges may non-specifically bind to positively-charged lipoplexes and rapidly remove them from the circulation via the reticulo-endothelial system [3]. Plasma proteins might also greatly alter lipoplex structure leading to aggregation and enhanced clearance or deterioration by serum nucleases. Opsonization and activation of the complement system [5] by lipoplexes are additional physiological phenomena that can participate in lowering the efficacy of intravenously (i.v.) administered cationic lipoplexes. Moreover, pharmacokinetic analysis of lipid-based delivery systems has revealed that, in the case of systemic administration, “first-pass” organs and those with a high degree of vascularization and blood flow (heart, lung, liver, spleen) exhibit significantly higher expression of the transfected molecule [2], [3], [6], [7] that is, consequently, cleared from the system very rapidly.

To overcome these drawbacks, great effort has been put into the formulation of stable cationic lipoplexes that can be successfully used *in vivo* [8]. We previously showed that lipoplexes with low net charge ratio exhibit higher gene or ON transfer following i.v. injection in almost all organs tested [2], [9]. Therefore, we designed a second generation vector system called Neutraplex (Nx) that allows the generation of negatively charged and stable lipoplexes [3], [10] thanks to the insertion of cardiolipin (CL) in the lipid composition. CL is a negatively charged phospholipid with specific chemical and biological properties that allow, first, the association (usually unstable) of lipids of opposite charge and, then, the elaboration of a versatile formulation to generate positively or negatively charged stable lipoplexes by only varying the DNA/lipid ratio [11]. Pharmacokinetic and bioavailability studies following the i.v. administration of an ON delivered either in free form, or using a cationic or a negatively charged Nx system in baboons [3] showed that Nx allowed to obtain high transfection efficiency with good distribution and slower clearance than classical cationic lipoplexes and without toxicity [3].

Another way to improve efficiency of ON transfection and activity would be to create molecules which can more easily enter the cells. However, despite several studies to investigate the mechanisms of uptake and intracellular trafficking of non-viral vectors to improve delivery, the current understanding of these processes is still limited. Endocytosis is generally considered to be the main entering pathway for lipoplexes [12]–[15]. Eukaryotic cells use several endocytic pathways to internalize a variety of substances and to accomplish different tasks: the clathrin-dependent pathway [16], phagocytosis [17], macropinocytosis [18] and the caveolin-dependent pathway [19]. Phagocytosis is restricted to specialized cells including macrophages, monocytes and neutrophiles [20]. Macropinocytosis is mainly used to internalize polyplexes, and less commonly for lipoplexes [21]. The type of endocytosis also depends upon the particle size [22]. In receptor-mediated endocytosis, small particles (<250 nm) are mainly internalized through the clathrin-mediated pathway, a non specific mechanism. This pathway is initiated by the formation of clathrin-coated pits that leads to the development of early and late endosomes, which ultimately fuse with lysosomes [23]. Larger particles (≥500 nm) generally enter the cells through caveolae [22], [23]. Although endocytosis has been proposed as the main pathway for lipoplex entry, a fusion mechanism has been also proposed in a few studies [24], [25]. An exchange mechanism could thus take place between lipoplexes and plasma membranes that would lead to destabilization of the lipoplex structure, DNA disassembling from the complex with delivery directly into the cell cytoplasm [1], [26].

Here we wanted to understand to which extent the surface charge of the lipoplexes influences the choice of cell internalization pathway. To this aim, we used the Neutraplex delivery system to prepare particles with the same lipid composition and structure but that are either positively or negatively charged. In this way we could directly compare their efficacy in delivering an ON into HeLa cells by analysing their uptake pathway, intracellular distribution and biological activity.

## Materials and Methods

### Cell line

HeLa cells stably transfected with the pLuc/705 plasmid (HeLa pLuc/705), in which the coding region of *luciferase* is interrupted by the mutated β-globin intron [28], were grown at 37°C, 5% CO_2_ in minimal essential DMEM medium (Gibco, Invitrogen SARL, Cergy Pontoise, France) supplemented with 10% foetal bovine serum (FCS), 1% Na pyruvate, non essential amino acids and a mix of penicillin, streptomycin and neomycin (100×, Gibco). Cells were checked every 3 weeks for the absence of mycoplasma contamination by using the PCR enzyme immunoassay mycoplasma detection kit (Roche Diagnostics).

### Antisense oligonucleotides

The 18-mer phosphodiester 2'O-methyl-oligoribonucleotides were purchased from Eurogentec SA (Seraing, Belgium). ON_705_ (CCU CUU ACC UCA GUU ACA) is the antisense (AS) sequence, targeted to an aberrant splice site that is created by a T to G mutation at nucleotide 705 of intron 2 of the β-globin in β-thalassemic patients [28]. ON_SC_ (ACU ACC CGA UAU CUC CUC) is the scrambled (SC) version of ON_705_. For fluorescence microscopy and flow cytometry, 5′-FITC- or 5′-Alexa_546_-labelled ON were used.

### Lipoplex formulations

The liposomes used for lipoplex (DLS and Nx) preparations consisted of small unilamellar vesicles (SUV) that can complex to negatively-charged ON. Neutraplex SUV were prepared by mixing 1 mg DOGS, 1 mg DOPE or FITC-labelled DOPE (Sigma-Aldrich Chimie SARL, St Quentin Fallavier, France) and 0.5 mg cardiolipin (CL, Sigma) in 50 µl ethanol and subsequently by adding excess pure water (6.25 mg lipid/mL, final) [3], [10]. Lipoplexes were obtained by mixing the SUV suspension with ON, at several lipid/ON weight ratios. Cationic (Nx+) and anionic (Nx−) lipoplexes were prepared, depending on the ON load charge in the formulation. Nx+20 or Nx−40 preparations corresponded to 20 or 40 µg ON with 156 µg total lipids, respectively. Typically, one Nx+20 preparation contained 128 µg ON/mg total lipids. DLS (Delivery Liposomal System) lipoplexes were prepared with DOGS and DOPE only, as previously described [29]–[31]. DLS/ON had a 10 µg ON to 185 µg total lipids ratio. DLS/ON preparation contained 54 µg ON/mg total lipids. Lipoplexes were stored at 4°C and used within 3 months from preparation.

### Particle size distribution and zeta potential analysis

Particle size and zeta potential were measured using a Malvern Zetasizer Nano ZS apparatus (Malvern Instruments, Orsay, France). All samples were kept at room temperature for 15 min prior to measurement and were analyzed undiluted. Sample analysis were carried out in automatic mode with the following parameters: scattering angle, 173°; temperature, 25°C; medium viscosity, 0.887 cP. Average diameters were evaluated as a Z-average using a monomodal method (NNLS cumulant analysis). Particle size data are expressed as hydrodynamic diameter vs. intensity. Coefficient of variation of our mean particle measurement under the experimental conditions is 15%. Zeta potential measurements were carried out using the M3-PALS technology. Width at half peak height is indicative of the homogeneity of size and charge distribution.

### Transfections and reporter gene assays

3.5×10^5^ HeLa pLuc/705 cells/well were seeded in 6-well dishes with 10% FCS-containing medium. After overnight culture (16–20 h), cells were rinsed twice with PBS and the medium was replaced with the OptiMEM medium or the DMEM medium containing decomplemented FCS (Invitrogen). OptiMEM (Invitrogen) is a supplemented synthetic medium. Lipoplexes were then added to the cells and luciferase activity was monitored 24 h later. Cells were rinsed twice with PBS and 300 µl Reporter Lysis Buffer (Promega France, Charbonnieres) was added. Protein concentration in the extracts was measured with the BCA™ Protein Assay Kit (Pierce, Perbio Science France, Brebieres) at 560 nm. Firefly luciferase activity was measured using a Luciferase Assay Kit (Promega) and was quantified in the supernatant with a Berthold Centro LB 960 luminometer (Berthold France SA, Thoiry). Luciferase activity in cultured cells was expressed as relative light units (RLU) per µg of protein. Each data point was averaged over three replicates.

### RT-PCR analysis of splice correction

After carrying out the luciferase and BCA™ Protein assays, cell lysate leftovers (about 270 µl) were transferred into 2 mL microfuge tubes and total RNA was extracted with 1 mL TRI Reagent (Sigma), according to the manufacturer's instructions. Specifically, 0.3 mL of chloroform was used for extraction and the amount of isopropanol for RNA precipitation was increased to give a 1∶1 mixture with the aqueous phase. RNA was quantified by RT-PCR amplification (MJ Research PTC200 Peltier Thermal cycler) with 5′ TTG ATA TGT GGA TTT CGA GTC GTC 3′ (forward primer) and 5′ TGT CAA TCA GAG TGC TTT TGG CG 3′ (reverse primer) and the SuperScript One-Step RT-PCR System with Platinum Taq (Invitrogen). Products were run on a 2% agarose gels, scanned and analyzed using the Gene Tools Analysis Software (SynGene, Cambridge, UK).

### Flow cytometry

5×10^5^ HeLa pLuc/705 cells/well were plated in 6-well dishes. After overnight culture, cells were incubated with lipoplexes containing 5′-FITC-labelled ON_705_ in OptiMEM or in DMEM with decomplemented serum at 37°C or 4°C (by keeping cells on ice) for evaluating the contribution of the temperature-dependent or -independent uptake (TDU or TIU, respectively). After 1, 3 or 6 h incubation, cells were rinsed twice with cold PBS, centrifuged at 4°C, resuspended in 300 µl cold PBS, in polystyrene tubes, and kept on ice until analysis. Cells to be transfected at 4°C were pre-incubated at this temperature for one hour. To strip-off lipoplexes bound at the cell surface, cells were treated with 1 mL glycine (0.2 M, pH = 2.8) for 30 min at 4°C and rinsed in ice cold PBS before FACS analysis [26], [27]. We determined the fluorescence intensity values corresponding to: FA, the fluorescence intensity of cells incubated at 37°C; FB, the fluorescence intensity of cells incubated at 4°C; FC, the fluorescence intensity of glycine- treated cells. We calculated:

- cell surface bound ON = FA-FC,
- TIU = FB-cell surface bound ON,
- TDU = FA-TIU-cell surface bound ON.

Flow cytometry was performed with a FACS Canto (BD Biosciences France, Le Pont De Claix, France). Fluorescence intensity of 10,000 events was recorded at 520 nm after excitation at 488 nm. Results were analyzed with the WinMDI Software. Each experiment was performed in duplicate and each data point was averaged over two replicates.

### Live cell observation by fluorescence microscopy

5×10^5^ HeLa pLuc/705 cells/well were plated in 35 mm dishes. Following overnight culture, cells were treated with lipoplexes with 5′-FITC-labelled-DOPE (Sigma) or 5′-Alexa_546_-labelled ON_705_ (Eurogentec), in 10% FCS-containing medium. Living cells were directly observed in the dish with 1 mL of 10% FCS-containing medium, after 1 to 24 h of transfection. Fluorescence distribution was analyzed on a Zeiss Axiovert 200 M fluorescence microscope without fixation (Carl Zeiss, Oberkochen, Germany), with an ×63 objective. Images were first acquired with the AxioVision software and, after exportation, they were analyzed with Adobe Photoshop and ImageJ.

### Cytotoxicity assay

Cell viability and cytotoxicity studies were performed by plating pLuc/705 HeLa cells in 96-well plates at 3×10^4^ cells/well. Cells were incubated with lipoplexes containing different ON concentrations for 24 h in OptiMEM or in DMEM with decomplemented serum. The proportion of live cells was determined using the tetrazolium-based colorimetric cell proliferation assay (MTS, Promega) by reading the absorbance at 560 nm. Data are expressed as the percentage of cytotoxicity (relative to untreated cells).

### Statistical analysis

Data were expressed as mean±standard deviation. Statistical analyses were performed using Student's *t* test for comparison of means. A probability of less than 0.05 was considered to be statistically significant.

## Results

### Physico-chemical properties and cytotoxicity of lipoplexes with different charges

To assess whether the surface charge of lipoplexes could influence the choice of cell internalization pathway we used two different formulations of liposomes to prepare lipoplexes: DLS that allows to obtain cationic lipoplexes with a low net charge ratio and Nx to prepare positively or negatively charged lipoplexes. First, we compared the physico-chemical properties of the different liposomes and lipoplexes by measuring the particle size range by Dynamic Light Scattering and the surface charge by zeta potential (Table 1). Homogeneous lipid particles were obtained with both formulations (Data S1). No particles larger than 600 nm were detected in any formulation. The global net charge ratios of positive charges in DOGS to negative charges in cardiolipin (CL) and ON were calculated. Global net charge ratios of 1.8 for Nx+20 and 1.0 for Nx−40 were obtained, suggesting that Nx−40 is the lipoplex preparation closest to neutrality. In comparison, we obtained a ratio of 7.3 with the DLS formulation. ON are fully protected in DLS or Nx lipoplexes when incubated for 4 hours in serum with medium as assayed by electrophoresis (PAGE) (data not shown). PAGE and cryo-electron microscopy analysis revealed that no excess material was detected in Nx lipoplexes containing 10 to 40 µg ON. As formation of complexes with 25–30 µg ON lead to global net charge neutralization and particles aggregation, we decided to use Nx+20 and Nx−40, as the positively and negatively charged NxON.

**Table 1:** Physico-chemical characterization of the liposomes and lipoplexes used in this study.

TYPE OF FORMULATION		ON	SIZE, nm	ζ POTENTIAL (mV)
			mean (width)	
**LIPOSOMES**	DLS		137 (149)	N.D.
	Nx		170 (22)	N.D.
**LIPOPLEX**	DLS/ON	2'O-Me	179 (25)	+44
	Nx+20/ON	2'O-Me	278 (245)	+22
	Nx−40/ON	2'O-Me	203 (200)	−37

Particles mean diameter was measured by dynamic light scattering and surface charge by zeta potential. Particle size data are expressed as hydrodynamic diameter vs. intensity. Width, half peak height. Nx+, cationic Nx lipoplexes. Nx−, anionic Nx lipoplexes. Nx20 or 40, 20 or 40 µg ON mixed with 156 µg total lipids, respectively. N.D., not determined.

**Figure 1:**
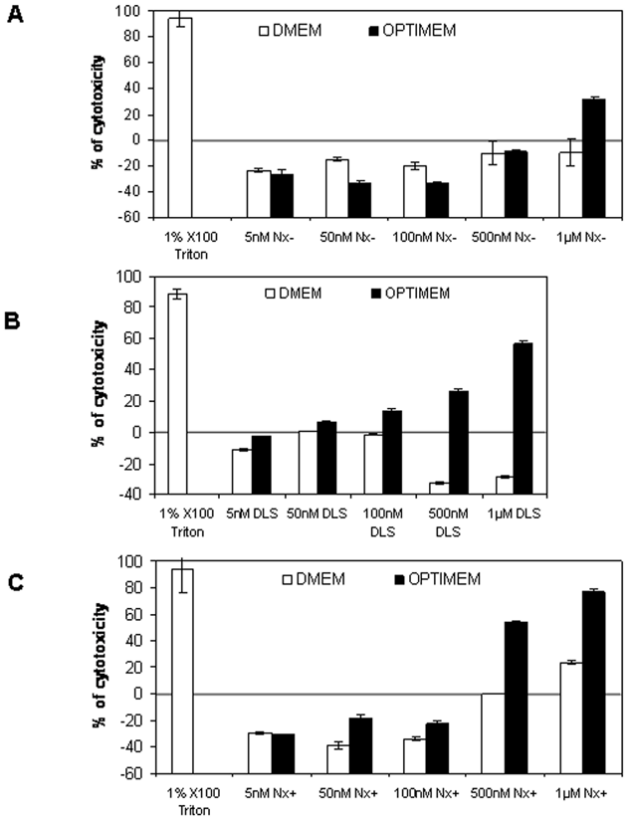
Cytotoxicity of increasing concentrations of ON_705_ delivered with Nx−40 (A), DLS (B), or with Nx+20 (C). HeLa pLuc/705 cells were incubated with the different formulations in serum-containing medium (white bars) or in optiMEM (black bars) for 24 h. ON concentrations complexed with the liposomes are indicated. The level of cytotoxicity was determined with the tetrazolium-based colorimetric cell proliferation assay and expressed as percent of the cytotoxicity level in treated cells in comparison to non-treated cells. Cells incubated with 1% X-100 Triton are used as positive control. Error bars show standard deviation (n = 3).

We then tested the cytotoxicity of our preparations by incubating HeLa pLuc/705 cells with DLS, Nx+ and Nx− lipoplexes containing different concentrations of ON_705_ in the presence (DMEM) or absence (OptiMEM) of serum and using a tetrazolium-based colorimetric assay. Cells grown in OptiMEM showed cytotoxicity starting from ON_705_ concentrations of 100 nM with DLS (Fig. 1B), of 500 nM with Nx− (Fig. 1A), and 1 µM with Nx+ (Fig. 1C) lipoplexes. No significant cytotoxicity was observed in cells grown in DMEM with the three formulations. Moreover, no decrease in protein concentration compared to untreated cells under these conditions was observed. As routinely observed when using DLS or Nx, higher cell growth was detected when using Lx low concentration explaining the “negative” cytotoxicity experimentally calculated when compared with non treated control cells (Fig. 1).

As a consequence, the concentrations of ON_705_ used for our study (i.e., lower than 250 nM) did not cause detectable cytotoxicity which could bias the interpretation of the results especially when assessing the uptake mechanism.

### Nx system internalizes ON

When using cationic lipoplexes, ON first concentrates in endocytotic vesicles, corresponding to a punctuate staining in the cytoplasm, and then, when they are free in cytoplasm, quickly accumulate in nuclei [29]–[31] and especially in nucleoli [31], [32]. After 6 h incubation, red fluorescence due to Alexa_546_-labelled ON_705_, delivered either with Nx+20 (A) or with Nx− 40 (B), was visible both in cytoplasm and in nuclei (arrows). Thus, the Nx system appears to promote ON internalization in HeLa cells as efficiently as more classical lipoplex formulations.

### Internalization mechanisms

**Figure 2:**
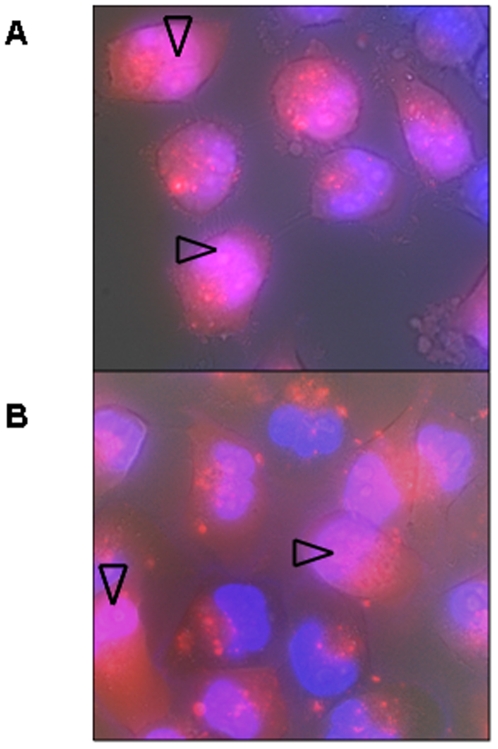
Epifluorescence images of unfixed HeLa pLuc/705 cells incubated with 200 nM Alexa_546_-labelled ON_705_ delivered with Nx+20 (A) or Nx−40 (B). Nuclei were stained with Hoechst (blue fluorescence) for 5 min. Living cells were directly observed under red and blue epifluorescence in PBS supplemented with 5% FCS, following 6 h incubation at 37°C. Intracellular ON appears in red and intranuclear ON in pink (arrows), whereas nuclei without ON are blue.

**Figure 3:**
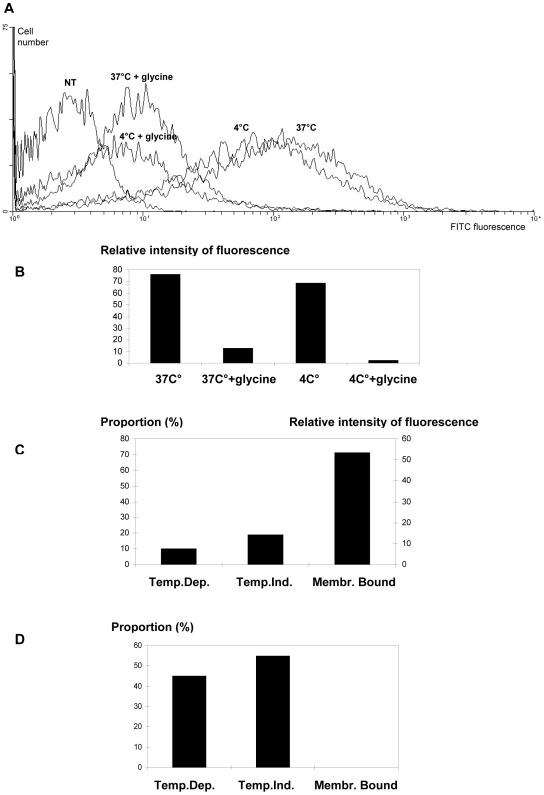
Evaluation of the contribution of temperature-dependent (TDU) or -independent uptake (TIU). FACS analysis data of HeLa pLuc/705 cells incubated with Nx+20 (A, B, C) or Nx−40 (D) lipoplexes are shown. Cells were incubated with 100 nM of 5′-FITC-labelled ON_705_ delivered with Nx+20 or Nx−40 lipoplexes. After 1 h incubation in optiMEM, at 4 or 37°C, Cells were rinsed with PBS and treated, or not, with glycine. ON binding to cell membrane was calculated by subtracting the fluorescence intensity of glycine-treated cells from that of non-treated cells. Estimation of TIU was determined as the fluorescence intensity of cells incubated at 4°C and treated with glycine buffer. Estimation of TDU was calculated by subtracting the sum of the fluorescence intensities corresponding to TIU and cell surface bound ON from the fluorescence intensity of cells incubated at 37°C. NT, non-treated cells. Two samples were used for each condition.

We then assessed the contribution of the different internalization pathways when lipoplexes with different electrical global net charges were used. Endocytosis, which is considered the main route used by lipoplexes, transports inert particles of mean diameter lower than 500 nm [21], [33]. Usually studies on particle cell uptake are performed by using conventional and specific markers of the late and medium endocytosis, however their respective contribution is not the purpose of our study. In addition, although endocytotic inhibitors such as chloroquine, NaN_3_, filipin or chlorpromazine are very helpful for studying drug intracellular release or elucidating a specific endocytosis route as we did in our laboratory for cell penetrating peptides [33], we decided to focus on plasma membrane penetration. Transport through plasma membrane mediated by lipid membrane fusion can not be specifically detected by specific inhibitors, such, for instance, fusion inhibitors like nigericin. Indeed, this ionophore highlights fusion events that only involve transmembrane pH gradients specific for instance of endocytic vesicles. For this reason, we focused our analysis on the thermo-dependence of the uptake mechanisms as endocytosis is an energy-dependent and temperature-dependent process and thus does not take place at 4°C. In order to assess the importance of endocytosis in the internalization of DLS, Nx+ and Nx− lipoplexes, we compared 5′-FITC-ON_705_ intracellular uptake at 1, 3 and 6 hours after transfection of cells kept at either 37°C or 4°C (Fig. 2), since inter-membrane lipid mixing is known to occur also at very low temperature [13], [15], [34], [35]. Moreover, in order to differentiate between non specific cell membrane binding and lipid fusion within the cell membrane, we incubated Nx-transfected HeLa pLuc/705 cells with glycine buffer (0.2 M, pH 2.8) to strip off the surface-bound ON prior to flow cytometry measurements [27], [32]. This treatment did not cause any cytotoxic effect in regards to microscopical examination, cell growth and splice correction efficacy within the time lapse between treatment and FACS analysis (data not shown). The efficacy of this treatment is demonstrated, for instance, by the observation that incubation with glycine of cells transfected with Nx+/ON_705_ for 1 h led to about 70% decrease in total ON-associated fluorescence (Fig. 3B and C). In addition, when cells were incubated with Nx-ON in OptiMEM culture medium for 1 h, subsequent glycine treatment did not decrease fluorescence intensity (Fig. 3D) demonstrating that glycine treatment did not interfere with the measurement of intracellular 5′-FITC-ON_705_.

**Figure 4:**
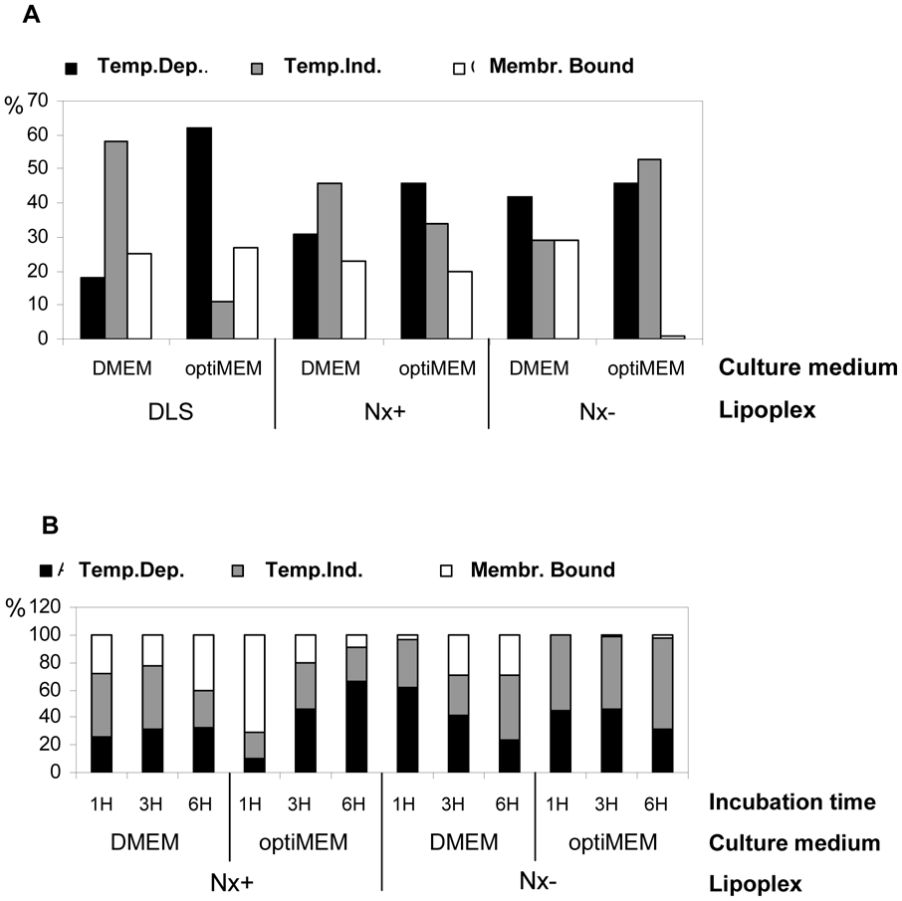
Estimation of the percentage (%) of temperature-dependent (TDU), independent uptake (TIU) and cell surface bound 5′-FITC-labelled ON_705_ delivered with DLS, Nx+20 or Nx−40 lipoplexes in HeLa pLuc/705 cells. Following 3 h (A) or 1, 3 and 6 h (B) incubation in serum-containing culture medium (DMEM) or OptiMEM, at 4 or 37°C, cells were rinsed with PBS and treated, or not, with glycine. Then, cells were subjected to flow cytometric analysis. Evaluation of ON fixation onto cells was calculated by subtracting the fluorescence intensity of glycine-treated cells from that of untreated cells. Evaluation of TIU was calculated by subtracting the fluorescence intensity corresponding to cell surface bound ON from the fluorescence intensity of untreated cells incubated at 4°C. Evaluation of TDU was calculated by subtracting fluorescence intensity corresponding to the sum of the cell surface bound ON and TIU fluorescence intensities from the fluorescence intensity of cells incubated at 37°C. Two samples were used for each condition.

When Nx+ was used to deliver 5′-FITC-ON_705_ in cells grown in OptiMEM (Figure 4B), temperature-dependent uptake (TDU) appeared to increase with time (from 8% after 1 h to 65% after 6 h of incubation, respectively) while surface-bound fluorescence decreased (from 71% to 7%). Temperature-independent uptake (TIU) fluctuated between 20 and 30% during the entire period of incubation. Conversely, in the presence of DMEM and serum, no or very little variation in the respective contribution of TDU, TIU and cell surface-bound was observed (from 25% to 31% for TDU, from 25% to 42% for TIU and from 22% to 41% for aspecific binding).

When Nx− was used to deliver 5′-FITC-ON_705_ in cells grown in OptiMEM (Figure 4B), no or vey little membrane-bound 5′-FITC-ON_705_ was detected, while TIU appeared to be the preferred uptake mechanism (from 55% after 1 h to 67% after 6 h of incubation). In the presence of DMEM and serum, however, 5′-FITC-ON_705_ showed mainly a TDU at the beginning (62% at 1 h) which then slowly decreased to 23% after 6 h of incubation, while TIU represent about 50% of the uptake at the same time point. Finally, overall cell uptake was higher with Nx+ than Nx− lipoplexes (4 and 2 -fold higher in optiMEM and DMEM, respectively). In conclusion, cationic lipoplexes exhibit the highest uptake level and favour TDU when cells are incubated in OptiMEM. TIU appears predominant when cells are treated in serum-containing medium. TIU and TDU are similarly used by anionic lipoplexes independently from the chosen incubation medium.

**Figure 5:**
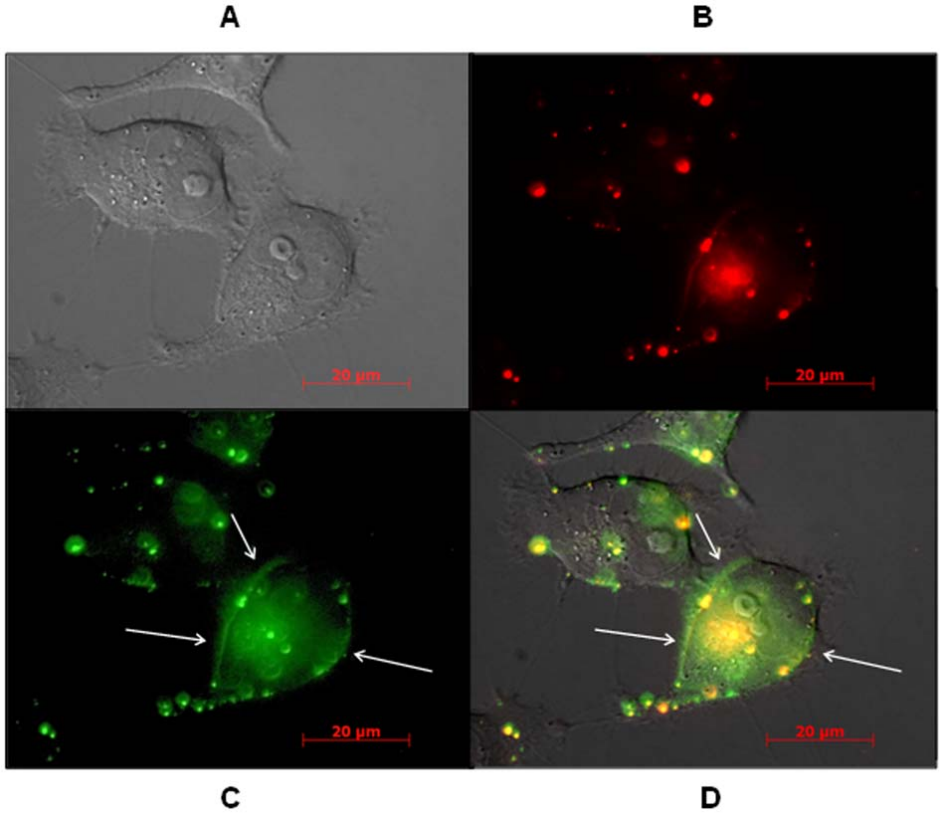
Epifluorescence images of unfixed HeLa pLuc/705 cells incubated with 250 nM Alexa_546_-labelled ON_705_ (red fluorescence) delivered with the Nx−40 system containing FITC-labelled lipids (green fluorescence). Living cells were directly observed in PBS supplemented with 5% FCS, after 1 h incubation at 37°C. (A), (B), (C) and (D) are images of the same field of observation. Images of cells were taken under visible light (A), red (B) or green (C) epifluorescence, and the three (A–C) images were then merged (D). Images are focused on cell membrane to observe lipid distribution consecutive to lipid exchange at the plasma cell membrane (white arrows).

We then followed by live microscopy the fate of Alexa_546_-labelled ON_705_ and of one of the lipids (FITC-labelled DOPE) of the Nx formulation under different experimental conditions. Figure 5 shows HeLa pLuc/705 cells grown in OptiMEM and incubated with Nx−40 and 250 nM of Alexa_546_-labelled ON_705_ at 4°C for 1 hr, i.e., experimental conditions that favour TIU (see Fig. 4). FITC-labelled DOPE (Figure 5C and Data S1, green) was both diffused in the cytoplasm and localized along the plasma membrane, suggesting fusion and displacement of the lipid within the membrane. Alexa_546_-labelled ON_705_ (Figure 5B, in red) was concentrated in endosomes and weakly distributed in the cytoplasm, probably due to low local concentration at this early time point. Partial nuclear localisation was observed after 6 hr of incubation but not after 1 hr of incubation (Figure 2).

### Splicing assay

**Figure 6:**
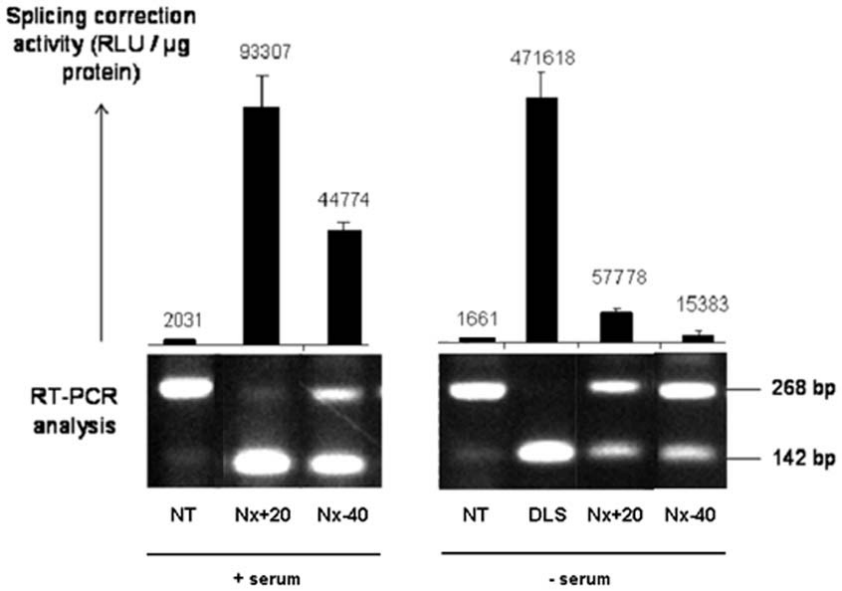
RT-PCR analysis of splice correction. HeLa pLuc/705 cells were incubated for 24 h in serum-containing-medium or OptiMEM, in the absence (NT) or in the presence of 100 nM ON_705_ delivered with the Nx or the DLS system. Cell samples were then divided in two. One fraction was lysed in lysis buffer and splicing correction was quantified by measuring luciferase activity as described in Material and Methods. The other cell fraction was subjected to total RNA extraction and amplification by RT-PCR. PCR products from incorrectly (268 bp) and correctly (142 bp) spliced samples were run on 2% agarose gels. Nx20 or 40, 20 or 40 µg ON mixed with 156 µg total lipids, respectively (surface charge, +22 and −37, respectively). DLS/ON has a 10 µg ON to 95 µg total lipids ratio (surface charge, +44).

In order to compare lipoplex efficacy in cell uptake and in delivering biologically active ON, we used a splicing correction assay. HeLa pLuc/705 cells were incubated for 24 hr with 100 nM ON_705_ in DLS or in Nx+20 and Nx−40 in DMEM and serum (Figure 6A) or OptiMEM alone (Figure 6B). When considering only the Nx formulation, Nx+20 appeared to be the most efficient formulation (Fig. 6). Splicing correction activity of Nx+20-ON_705_ was about twice than that of Nx−40-ON_705_. In dose curves experiments, incubation of cells in serum-containing medium with Nx+20 and Nx−40 led to a statistically significant difference in terms of luciferase activity compared to control (OD_SC_) at 100 nM (p<0.001) and 10 nM (p<0.01) (data not shown). ON_705_ delivered with DLS presented a 5–10 times lower activity in medium with serum (22,000 RLU/mg Prot., data not shown), but, as shown in Fig. 6, the DLS system was 8-fold more efficient than Nx+20 in the absence of serum.

## Discussion

In this work we show that: (i) it is possible to obtain homogenous and stable negatively or positively charged lipoplexes able to transfect cells, (ii) lipoplexes enter cells by either a temperature–dependent (TDU) or -independent mechanism (TIU), (iii) transfected ON activity depends on the type of lipoplexes used and the presence/absence of serum in the cell culture medium.

The study of the influence of particle charge of lipoplexes on cellular uptake or biological activity was made possible by the Neutraplex system which allows the formation of lipoplexes with either positive or negative charges and the same lipids (vector) composition [3], [10]. Lipoplexes formation is based on the Manning-Oosawa counter ion reaction that leads to compaction of nucleic acids (NA)/lipid complex which, under specific preparation processes, generate small and stable particles. To ensure complete DNA or ON counter ion condensation, excess of positive charges is usually needed to obtain stable complexes for most previously described lipoplex preparation, but for the Neutraplex system.

Data on the cytotoxicity of the different lipoplexes used in this study confirm that cationic lipoplexes are more toxic than the anionic ones. This effect is more pronounced when cells are incubated in OptiMEM than in serum-containing medium, highlighting the affinity of cationic lipoplexes for the cell surface and the fact that their binding with anionic serum proteins seems to strongly inhibit their cell uptake. Therefore, the strategy of using cationic lipoplexes to take advantage of interactions with the negative charges at the cell surface, such as in endothelial cells, appears restricted. In addition, the non-specific binding of positively charged particles to blood proteins greatly and rapidly neutralizes the net positive surface charge. Previous work has demonstrated the successful use of neutral or negatively charged liposomes to transfer the encapsulated plasmid DNA following systemic injection in rodents [35], [36]. These examples show that high plasmid DNA compaction and cationicity are not absolute requirements for successful gene transfer.

Formation of anionic Nx was made possible by the insertion of cardiolipin (CL) in the lipid composition. CL is a negatively charged phospholipid with particular chemical and biological properties that allow, first, the association (usually unstable) of lipids of opposite charge and, second, the elaboration of a versatile formulation generating positively or negatively charged, stable lipoplexes by only varying NA/lipid ratio [11]. Such Nx supramolecular assemblies are of spherical shape and exhibit a specific liquid crystal ultrastructure in which DNA is circumferentially packaged in a lipid bilayer [10]. CL does not specifically leads to this specific ultrastructure, as it was observed for DLS/DNA, but the presence of CL in Nx might facilitate the transition from lamellar to hexagonal phase [10], [11], [38]. CL is considered to favour the hexagonal ultrastructure and to enhance lipid membrane fusion. Indeed, we showed [38] that Nx/plasmid DNA particles have a higher proportion of hexagonal structures than DLS/plasmid DNA. The same Nx formulation with high molecular weight DNA (T4 phage DNA) led to mostly hexagonal structures as determined by cryo-electron microscopy and Small Angle X-rays diffraction [10], [38]. The emerging viewpoint is that structural changes of lipoplexes upon interaction with anionic cellular lipids may regulate lipofection efficiency [39].

Our work clearly demonstrates that TDU was the major uptake mechanism for cationic particles, especially for DLS and Nx+ in OptiMEM (62 and 66%, respectively for 3 and 6 h) like in previous studies [12]–[15]. However, TIU represented up to 62% for Nx− in serum-containing medium.

The high positive global net charge of DLS lipoplexes compared to Nx+20 (7.3 *versus* 2.0) might account for the higher contribution of TDU compared to TIU (2-fold compared to 0.6-fold) (Fig. 6A and B). This might be as well observed with commercially available cationic lipid systems or other systems with high global net charge ratio such as Lipofectamine (Invitrogene) (13.5). It has to be noted that Lipofectamine performs very poorly in splicing correction assay when used in serum-containing medium [29], [30].

Our results show that anionic particles can easily enter the cell despite the negatively charged proteoglycans located onto plasma membrane, which have been described as mediators for the uptake of cationic lipoplexes. Generally, increasing the negative charge in lipoplexes led to a decrease in membrane binding and a gradual decrease in TDU while increasing ON activity when serum is present in transfection medium. Conversely, increasing the positive charges led to an increase both of TDU and ON activity in optiMEM transfection medium. Membrane binding of cationic or anionic lipoplexes showed to increase with time in serum-containing medium, whereas it appeared fast and saturable when using OptiMEM.

These findings indicate that TIU might be, together with endocytosis, a significant pathway by which lipoplexes penetrate into the cell and release ON in the cytoplasm. We might hypothesize that TIU happens partly through lipid exchange at the cell membrane. Lipoplex fusion with inner membranes to promote ON release from endocytic vesicles following endocytosis was previously observed. Only few authors, reporting on highly efficient multi-component lipoplexes, have previously hypothesized that lipid mixing entropy may play a key role in the mechanism of internalization [40]. Several lipids (DOTAP, DOTMA, DOPE, CL) and polymers (PEG) that may facilitate lipoplex fusion with the plasma membrane [1], [3], [41]–[43] or with the endosome membrane [44] have been described. Nucleic acid delivery is efficiently achieved by viruses which penetrate through the plasma membrane by spontaneous merger of membranes promoted by fusion protein catalysts. A clear understanding of the thermodynamics and lipids rearrangements involved in this process remains incomplete [45]. Insertion of a fusion protein inside unilamellar liposomes was shown to promote lipid mixing and membrane fusion facilitating intracellular penetration of liposome content [45]. In various experimental models, it was established that synthetic membrane vesicles could merge by fusion of lipid membranes when using, in liposome composition, lipids supporting formation of the hexagonal H_II_ phase. Among those are phosphatidylethanolamine and cardiolipin which are constituents of the Nx lipoplexes. Large unilamellar vesicles containing cardiolipin undergo membrane fusion and lamellar-to-inverted hexagonal phase transition in presence of Ca^2+^ [46]. Non-leaky fusion of the vesicles over rapid collapse into H_II_ structures was observed at temperatures ranging between 0 and 50°C [34], [45]. Membrane fusion of a synthetic vesicle in live cells is poorly described [41]–[43]. Biological membrane fusion is a localized, fast (less than a millisecond) and a well controlled (non leaky) process. It could be assumed that anionic lipids laterally diffuse in the lipoplex membrane surface neutralizing the cationic lipids in a flip-flop process and consequently release ON as postulated by Zelphati and Szoka for the endosomal release [14]. H_II_ phase have been observed by electron microscopy in both prokaryotic and eukaryotic membranes but various aspects of biomembranes heterogeneity during membrane fusion are not fully elucidated. As a consequence, it is conceivable to hypothesize that lipid exchange might occur at 4°C and might contribute to the intracellular transport of ON at higher temperature. We observed that high TIU is correlated with low levels of cell surface bound ON, in particular when delivered with Nx−. We hypothesize that lipid fusion is a fast process or/and that cell surface proteoglycans might maintain lipoplexes at the plasma membrane holding back penetration. We suggest that TIU, observed when using the Neutraplex lipoplexes, might be due to lipid mixing and concomitant membrane fusion. Certainly, the use of cardiolipin in presence of a cationic compound and the specific highly ordered ultrastructure promoting lamellar to hexagonal phase transition of Nx [10] could be important factors to facilitate lipid mixing with plasma or endocytic vesicle membranes and release of free ON in the cytoplasm. A fusion mechanism bypasses the vesicular endocytic traffic avoiding lysosome hydrolytic enzymes and, therefore, could lead to higher ON release. This pathway could be more efficient compared to endocytosis in some conditions. Cell lines develop high endocytic activity with consecutive cell culture passages; therefore, the use of high passage cells in combination with lipoplexes of high net global charge ratio certainly led to the assumption that endocytosis is the main uptake mechanism. It has to be noted that primary cells or haematopoietic cells, such as peripheral blood mononuclear cells, are difficult to transfect possibly due to their poor ability to elicit endocytosis. We also observed that mesenchymal stem cells promoted lipid exchange mechanism to take up lipoplexes (data not shown). We believe that the possibility of a fusion mechanism should be taken into consideration when developing lipoplexes designed for *in vivo* or *ex vivo* nucleic acid transfer.

Interestingly, ON delivered with Nx lipoplexes are more active that ON delivered with formulations close to neutrality while precluding aggregation (conventionally observed when preparing neutral membranes particles). This suggests that high positive global net charge for lipoplexes is not a requirement for cell transfection in contrast to the conclusions drawn from the literature [7], [13]–[15]. Thus, this observation might contribute to pave the way for the development of less toxic and more efficient lipoplexes.

Here we demonstrated that it is possible to obtain very good splicing correction with negatively charged lipoplexes in serum-containing medium at ON concentrations as low as 10 nM [30] and data not shown] which is one of the lowest effective concentrations described in the literature [47]–[50]. Pharmacological considerations and delivery route are important when considering splice switching ON for therapeutic use [48]–[50]. Although lipoplex formulations might increase treatment cost and technological requirements, they might compensate these drawbacks by making possible the use of potentially less toxic, non modified splice switching ON with higher activity and specific biodistribution. Controlling ON/lipoplex uptake cell mechanism by design might influence cell targeting following systemic or local administration and *ex vivo* ON strategies.

## Supporting Information

Data S1 (3.64 MB DOC)
