# Supplementary material for: Physico-Chemical Characteristics of Lipoplexes Influence Cell Uptake Mechanisms and Transfection Efficacy
Source: PLoS One. 2009 Jun 26;4(6):e6058. doi: 10.1371/journal.pone.0006058 (PMC2699663; doi:10.1371/journal.pone.0006058)
Supplement: Data S1 — (3.64 MB DOC) [file pone.0006058.s001.doc]

**supplementary data #1.** Lx size distribution as determined by Dynamic Light Scattering. Particle size data are expressed as hydrodynamic diameter vs. intensity. Nx+, cationic Nx lipoplexes. Nx-, anionic Nx lipoplexes. Nx20 or 40, 20 or 40 µg ON mixed with 156 µg total lipids, respectively. (A), DLS Lx; (B), Nx+20; and (C), Nx-40.

**supplementary data #2.** Epifluorescence images of unfixed HeLa pLuc/705 cells incubated with 250 nM ON_705_ delivered with the Nx‑40 system containing FITC-labelled lipids (green fluorescence). Living cells were directly observed in PBS supplemented with 5 % FCS, after 1 h incubation at 37°C. (A, B), (C, D), and (E, F) are images of the same field under visible light (A, C, E) or green epifluorescence (B, D, F).

**SUPPLEMENTARY DATA #1**


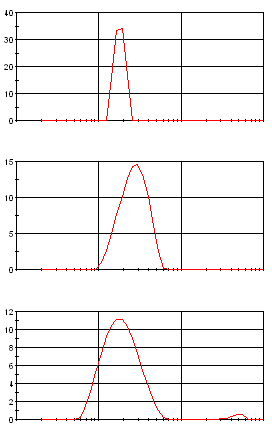


**Intensity (%)**

10 100 1000 10,000

10 100 1000 10,000

10 100 1000 10,000

**A**

**B**

**C**

**Diameter size (nm)**

**SUPPLEMENTARY DATA #2**

**A C E**

**B D F**
